# Impact of Melt Processing Conditions on the Degradation of Polylactic Acid

**DOI:** 10.3390/polym14142790

**Published:** 2022-07-08

**Authors:** Thamer Aldhafeeri, Mansour Alotaibi, Carol Forance Barry

**Affiliations:** Department of Plastics Engineering, University of Massachusetts Lowell, Lowell, MA 01854, USA; thamer_aldhafeeri@student.uml.edu (T.A.); mansour_alotaibi@student.uml.edu (M.A.)

**Keywords:** polymer degradation, twin screw extruder, quad screw extruder

## Abstract

To reduce the degradation of polylactic acid (PLA) during processing, which reduces the molecular weight of PLA and its properties, prior studies have recommended low processing temperatures. In contrast, this work investigated the impact of four factors affecting shear heating (extruder type, screw configuration, screw speed, and feed rate) on the degradation of PLA. The polylactic acid was processed using a quad screw extruder (QSE) and a comparable twin screw extruder (TSE), two screw configurations, higher screw speeds, and several feed rates. The processed PLA was characterized by its rheological, thermal, and material composition properties. In both screw configurations, the QSE (which has a greater free volume) produced 3–4 °C increases in melt temperature when the screw speed was increased from 400 rpm to 1000 rpm, whereas the temperature rise was 24–25 °C in the TSE. PLA processed at low screw speeds, however, exhibited greater reductions in molecular weight—i.e., 9% in the QSE and 7% in the TSE. Screw configurations with fewer kneading blocks, and higher feed rates in the QSE, reduced degradation of PLA. At lower processing temperatures, it was found that an increase in melt temperature and shear rate did not significantly contribute to the degradation of PLA. Reducing the residence time during processing minimized the degradation of PLA in a molten state.

## 1. Introduction

The large quantity of plastic waste produced by non-degradable polymers is a global concern [1]; therefore, it is crucial to utilize sustainable and biodegradable replacements for petroleum-based polymers. Sustainable polymers are derived from renewable resources. Greater use of sustainable polymers reduces dependence on fossil fuel-based resources [1,2,3]. In addition, many sustainable polymers, including polylactic acid, poly-3-hydroxybutarate, polycaprolactone, and naturally occurring polymers are biodegradable, which provides opportunities to reduce plastic waste. Some sustainable and biodegradable polymers have mechanical properties that are comparable to those of petroleum-based plastics. Their biodegradability, however, makes them more difficult to melt process. For example, commercially available biodegradable aliphatic polyester polymers exhibit a wide range of properties and are competitive with non-biodegradable polymers in a variety of applications [4]. The ester linkages present in these aliphatic polyesters are susceptible to chemical hydrolysis as well as enzymatic chain cleavage. These characteristics make aliphatic biodegradable polymers sensitive to moisture and heat during melt processing [5].

A promising alternative to petroleum-based polymers is the biodegradable polymer, polylactic acid (PLA) [1,2,3,4,5]. Polylactic acid is a linear aliphatic thermoplastic polyester, manufactured from non-toxic monomers; these monomers are produced from renewable natural resources such as starch and sugar [6,7,8]. Depending on the stereopurity of its backbone, it can be either semi-crystalline or amorphous [9]. Although the molecular weight and stereochemical composition of PLA influence its mechanical properties, PLA can exhibit high modulus and high strength [10]. These properties make PLA a promising candidate to replace conventional polymers like polyolefins in consumer goods, packaging, and other applications [10,11,12]. PLA, however, still has some serious drawbacks that limit its applications. One issue is that PLA is susceptible to thermal degradation during melt processing [3,4,5,6,7,8]. Any significant thermal or thermo-mechanical loads produce random chain scission as one of the predominant degradation pathways for PLA [13]. This degradation process leads to the formation of cyclic polylactic acid oligomers and lactic acid, which is catalyzed by the presence of monomers, oligomers, and hydroxyl groups [14]. These degradation mechanisms produce significant reductions in molecular weight and narrowing of the molecular weight distribution, which deteriorates the mechanical properties, changes the rheological and crystallization behavior, and causes yellowing of the processed polymer [15,16,17]. In addition, other drawbacks of PLA include poor gas barrier properties, low toughness and ductility, and high cost [18,19].

The degradation of polylactic acid during melt processing has been evaluated using rheological measurements, differential scanning calorimetry (DSC), and Fourier-transform infrared radiation (FTIR) [20,21,22]. Reductions in molecular weight reduce the melt viscosity. The zero-shear viscosity, which is measured at low shear rates, provides a better indication of degradation because alignment of the polymer chains at high shear rates also reduces the melt viscosity [20]. Reductions in molecular weight provide extra mobility to the polymer chains, which leads to an increase in crystallinity [21]; this crystallinity can be measured using DSC. Patti et al. [22] assessed the degradation of polylactic acid using an FTIR characterization technique. They evaluated three absorption bands associated with the scission of esters in the polymer chains: 1750 cm^−1^, which is linked to carbonyl (C=O) stretching, 1183 cm^−1^, and 1085 cm^−1^, both of which are attributed to the asymmetric vibration of the ester group (C–O–C).

Several research groups have evaluated the effect of melt temperature on the degradation of polylactic acid during twin screw extrusion [20,22,23]. Mysiukiewicz et al. [20] assessed the deterioration of multiple grades of polylactic acid due to processing temperature. Regardless of the PLA grade (i.e., molecular weight of the virgin resin), the results indicated that higher processing temperatures significantly lowered the molecular weight of the polylactic acid. For example, the zero-shear viscosity of polylactic acid, with a melt index of 10 g/10 min, decreased from 1844 Pa.s to 119 Pa.s when the processing temperature was increased from 180 °C to 260 °C at 50 rpm. When Taubner and Shishoo [23] studied the influence of processing temperature on the degradation of poly (L-lactide), they concluded that the processing temperature must be kept at a low level to diminish the degradation of polylactic acid during the process. Please note that processing with a miniaturized internal mixer also created a significant reduction in molar mass as the processing temperature was increased from 170 °C to 210 °C [24].

Researchers have also investigated the degradation of PLA using very low screw speeds (25 rpm to 250 rpm) and low processing temperatures. Taubner and Shishoo [23] stated that a reduction in the average molecular weight of PLA was less important than residence time at a lower processing temperature (210 °C). When Mysiukiewicz et al. [20] evaluated the deterioration of multiple grades of polylactic acid using low screw speeds (50, 100, 150, 200, and 250 rpm), they stated that processing polylactic acid at higher screw speeds showed better results. The reported data, however, showed higher degradation of PLA in some grades when the screw speed was 250 rpm. Although screw speed, screw configuration (design), and feed rate (which affects the fill level and the shear) contribute to viscous dissipation during twin screw extrusion, there has been no investigation of the effects of screw configuration and feed rate on the degradation of PLA.

Most commercial compounds of biodegradable polymers, such as PLA, occur in twin screw extruders (TSEs) [11,20]. Other continuous mixing equipment, however, may offer opportunities for less degradation of PLA. One system is a quad screw extruder (QSE), which contains four parallel corotating screws. The QSE has a greater free volume, which reduces the shear applied to the polymer and creates lower increases in melt temperature. With polyethylene, Albareeki et al. [25] found that increasing the screw speed from 400 rpm to 2000 rpm produces a 65 °C increase in melt temperature for a TSE, but only a 15 °C rise in melt temperature for a QSE (both the TSE and QSE had the same diameters, length-to-diameter ratios, and screw configurations). The greater free volume in the QSE also permits greater throughput than in a TSE.

This work investigated the impact of four factors affecting shear heating (screw speed, screw configuration, extruder type, and feed rate) on the degradation of polylactic acid. Higher screw speeds were used to determine the balance between shear rates and shearing time (i.e., extruder residence time). Two screw configurations provided the effect of kneading blocks compared to conveying elements. The impact of greater free volume in a QSE was compared to the tighter spacing in a similar TSE. Feed rates for the QSE were increased to evaluate the effects of greater filling of the screw channels and reduced shear on the PLA. Following this, degradation was measured by studying changes in zero-shear viscosity, melt index, chemical composition, and crystallinity of the PLA. The results unexpectedly contradicted prior assumptions about viscous dissipation in the extruders.

## 2. Materials and Methods

### 2.1. Materials

This work was conducted using commercial grade polylactic acid (Nature Works LLC, grade: Ingeo^TM^ Biopolymer 2003D, USA, Minnetonka, WI, USA). This PLA is considered to be of “general purpose extrusion” grade. The reported density was 1240 kg/m^3^ and melt flow rate (210 °C/2.16 kg) was 6.0 g/10 min. Prior to extrusion, the PLA was dried using a desiccant drier (Dri-Air Industries, model: MPD-30D, Windsor, CT, USA) at a temperature of 55 °C for 24 h to prevent hydrolysis. The moisture levels in the dried PLA were measured using a moisture analyzer (Computrac, model: Vapor ProXL, Chandler, AZ, USA); these levels were consistently 0.021% which was within the supplier-recommended range of <0.025%.

### 2.2. Processing

**Figure 1:**
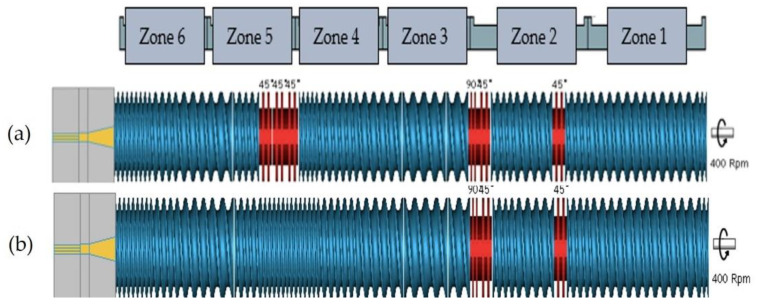
Screws for the TSE and QSE: (**a**) screw configuration 1 with kneading blocks in the mixing zone (zone 5) and (**b**) screw configuration 2 with only conveying elements in zone 5.

To evaluate the impact of the greater free volume and greater screw intermeshing in a QSE compared to a TSE, the extrusion of PLA was performed using a TSE (Technovel Corporation, model: KZW15 TW, Osaka, Japan) and a QSE (Technovel Corporation, model: WDR15QD, Osaka, Japan). Both extruders had 15-mm diameter screws, length-to-diameter ratios of 45:1, and the same screw configurations. The two screw configurations are shown in Figure 1. Both screw configurations contained one set of 90° kneading blocks (KBs) and 2 sets of 45° forward KBs in the melting section (zone 2). The KBs were required to melt the PLA. As illustrated in Figure 1a, screw configuration 1 also had three sets of 45° forward KBs in the mixing zone (zone 5). In contrast, screw configuration 2 contained only conveying elements in zone 5 (Figure 1b). The use of only conveying elements in the mixing zone helped to determine how the three-intermeshing region contributed to the increase in melt temperature and to the degradation of PLA.

The PLA pellets were fed at the beginning of zone 1. Melt exiting the extruders passed through a single strand die in the TSE and a double strand die in the QSE. The extruded strands were cooled in a water bath and then pelletized (Bay Plastics Machinery Company LLC, Bay City, MI, USA). The processing and equipment used for the extrusion trials are listed in Table 1. In both trials, the barrel and die temperatures were held constant at a relatively low temperature to avoid degradation of the PLA; these temperatures were based on recommendations from prior studies. Trial 1 examined the effect of screw configuration and screw speed in the TSE and QSE using the maximum feed rate permitted by the TSE (i.e., 2 kg/h). The screw speed of 400 rpm was typical of prior work in TSEs and the screw speed of 1000 rpm was near the maximum screw speed allowed by standard TSEs. Trial 2 investigated the effect of feed rate in the QSE using screw configuration 2 and the lower screw speed of 400 rpm. Higher feed rates reduced shear rates in the extruder, and preliminary work had shown that the QSE had sufficient free volume and drive torque for feed rates of 2, 3, and 4 kg/h. In Trials 1 and 2, extrusion with each set of specific parameters was performed and data were collected for several hours after the extrusion process was stabilized; the drive torque, barrel and die temperatures, and head pressure were monitored to ascertain process stability.

Melt temperature was measured using a type K immersion thermocouple (Dynisco Inc., Franklin, MA, USA), by extending 6 mm into the melt stream which was located in the die adaptors. Although immersion thermocouples produce somewhat high melt temperature readings due to shear heating, this thermocouple provided a relative measurement of the increase in melt temperature associated with changes in extrusion parameters. Residence time was determined using a pigment as a tracer. Multiple melt temperature measurements were recorded during extrusion and these values were averaged. This pigment was fed directly into the feed port with the PLA pellets, and residence time was measured as the time required to detect visual changes in the color of the extrudate.

### 2.3. Characterization

#### 2.3.1. Rheological Properties

To evaluate the degradation, the dynamic rheological characterization in the molten state of PLA was accomplished using a parallel plate rheometer (TA Instruments, model: ARES-G2, Newcastle, DE, USA). The plates had diameters of 25 mm and a gap of 1.5 mm. The specimen disks were prepared by micro-injection (Xplore Instruments BV, model: M-12, Sittard, The Netherlands) with a soak time of 3 min and a barrel temperature of 180 °C. Prior to the molding and the rheology trials, the samples were dried in a vacuum oven at 55 °C for 24 h. The rheological characterization was carried out at 180 °C. A strain sweep was used to verify that the strain used in frequency sweeps was within the linear viscoelastic region. Frequency sweeps were then conducted from 0.02 to 15.92 Hz at 2% of strain while recording the complex viscosity (η*). To ensure accurate results, 2 replicates were conducted for each sample and the data are presented as the mean ± the standard deviation.

Since polylactic acid generally obeys Cox–Merz rules [26], the complex viscosities and angular frequencies were considered equivalent to shear viscosities (η) and shear rates ($\dot{\gamma })$, respectively. In each processing condition, the zero-shear viscosity (η_o_) and relaxation time (λ) were determined using Trios 5.0 software (TA Instruments, Newcastle, DE, USA) to fit the viscosity–shear rate data to the Carreau–Yasuda model [27]:

(1) $$\eta \left(\dot{\gamma }\right)=\eta_{o}{\left[1+{\left(\lambda \;\dot{\gamma }\right)}^{a}\right]}^{\frac{n-1}{a}}$$

where *n* is a power-law coefficient, *a* is the adjustable exponent, and λ is the relaxation time.

#### 2.3.2. Melt Flow Index

The melt flow index (MFI) measurements were performed in accordance with ASTM D1238-20, Procedure A, using a temperature of 210 °C and load of 2.16 kg. Five measurements were carried out in the extrusion plastometer (Dynisco Inc., model: 710-1-5-010-14, Franklin, MA, USA) and the average values were calculated. The ratios of average MFI of the reprocessed sample to the average MFI of the virgin sample (MFI/MFI_o_) were reported.

#### 2.3.3. FTIR

**Figure 2:**
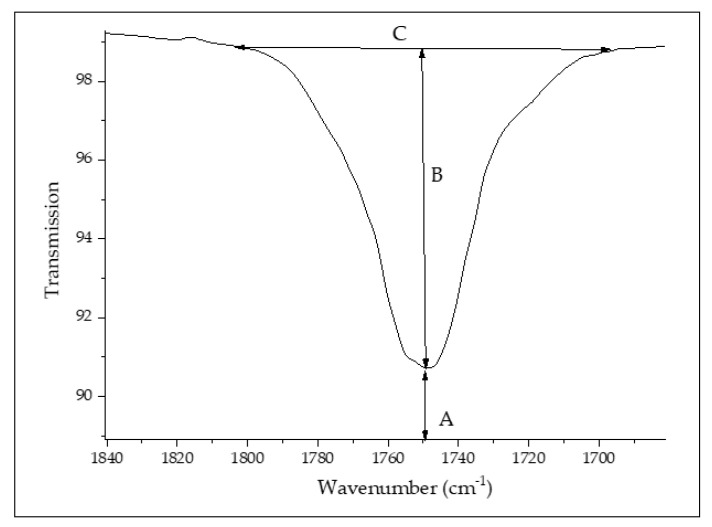
Method used to calculate the peak ratios adapted from [28].

Fourier-transform infrared (FTIR) spectra of the virgin and processed PLA from Trials 1 and 2 were obtained using an FTIR spectrometer (Nicolet™ iS50, Thermo Fisher Scientific, Waltham, MA, USA) in ATR mode. The room temperature measurements were performed at 4 cm^−1^ resolution and 64 scans. The test specimens were small pieces of pellet. To ensure accurate results, at least 2 replicates were conducted for every sample; the data are presented as average values with standard deviations. The degradation of PLA was assessed using absorption bands at 1750 cm^−1^ (which is associated to carbonyl (C=O) stretching), 1183 cm^−1^, and 1085 cm^−1^ (which are linked to the asymmetric vibration of the ester group (C–O–C)) [22]. The peak height ratios were determined using the mathematical expressions proposed by Sabnis and Block [28]:

(2) $$\mathrm{Peak}\;\mathrm{height}\;\mathrm{ratio}=\frac{H_{\mathrm{Peak}\;X}}{H_{\mathrm{Peak}\;\mathrm{Ref}}}$$

where H_Peak X_ and H_Peak Ref_ are the heights of the study peak and reference peak, respectively (1455 cm^−1^). These peak heights were calculated using:

(3) $$H_{\mathrm{peak}}=\log_{10}\frac{\mathrm{AC}}{\mathrm{BC}}$$

where AC is the height for the base line and BC is the absolute height of the absorption band of the functional groups at the respective wavenumber (Figure 2). These peak areas were determined using OriginPro 8.1 software (OriginLab Corporation, Northampton, MA, USA).

#### 2.3.4. Differential Scanning Calorimetry

Differential scanning calorimetry (DSC) was used to determine changes in thermal behavior of the processed polylactic acid. The tests were carried out using a Mettler Toledo instrument (model: DSC 3+, Columbus, OH, USA). Material samples of approximately 5.0 to 9.0 mg were placed in aluminum crucibles with pierced lids. In an inert nitrogen atmosphere, these crucibles were heated to 200 °C at a rate of 10 °C/min, held in the molten state for 5 min, and then cooled to 20 °C at a rate of 10 °C/min. The heating/cooling cycle was repeated twice to erase the thermal history of the material and to evaluate the DSC curves from the second melting step. Calculations employed a melting enthalpy of 93.0 J/g reported for 100% PLA [29]. STARe software (Mettler Toledo) was used to determine the glass transition temperature (T_g_), melt temperature (T_m_), and percent crystallinity of the virgin and processed PLA samples.

## 3. Results and Discussion

### 3.1. Extrusion Parameters

**Figure 3:**
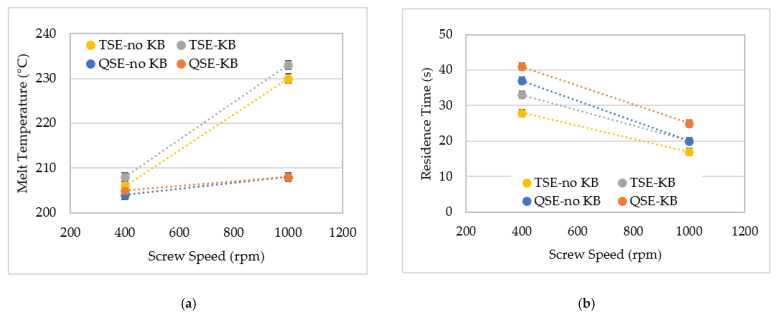
The effect of screw speed on (**a**) melt temperature and (**b**) residence time for the TSE and QSE, with a feed rate of 2 kg/h.

Figure 3a presents the effect of screw speed and screw configuration on the average melt temperature recorded for the constant feed rate of 2 kg/h. At 400 rpm, both the TSE and QSE extruders and both screw configurations showed a temperature difference of only 4 °C. These results are consistent with previous studies where TSEs were operated at relatively low screw speeds (250–500 rpm) and with several screw configurations [30,31]. For instance, Farahanchi and Sobkowicz [31] reported a 5 °C increase in temperature when the 15-mm-diameter TSE had three screw configurations with different amounts of kneading blocks. As expected, the melt temperature increased with increasing screw speed. At the higher screw speed of 1000 rpm, the melt temperatures depended on the extruder type. The QSE produced 3 °C to 4 °C increases in temperature, whereas the temperature rise was 24 °C to 25 °C for the TSE. The temperature differences between the TSE and QSE were consistent with the calculated free volumes, which are 165 cm^3^ and 273 cm^3^, respectively [25]. The greater free volume in the QSE reduced the shear and the resultant viscous dissipation. In contrast, high impact friction occurs at the interfaces between the polymer-barrel wall and the polymer screw, as melt in twin screw extruders flows in a tight figure-eight pattern [25,32]. Due to the tight clearances in the TSE, the polymer melt undergoes high levels of shearing [33]. The presence of 45° forward kneading blocks in the mixing zone had little effect on melt temperature, because forward kneading blocks have higher positive conveyance and provide less filling of screw elements in comparison to neutral or reverse kneading blocks.

As shown in Figure 3b, residence time decreased with increasing screw speed, and the residence times for the QSE were about 20% higher than for the TSE. The higher residence times of QSE were due to the greater travel distance of melt inside the barrel. In addition, according to the flow patterns for high and low screw speeds in the twin screw extruder, it was assumed that the circumferential flow lengths with high screw speeds were greater than those with low screw speeds, as the radii of flow circulation are larger and the melt temperature is higher at high screw speeds [34]. The kneading blocks in the mixing zone produced a 3 to 5 s increase in residence time because forward kneading blocks do not convey material as rapidly as conveying elements.

**Figure 4:**
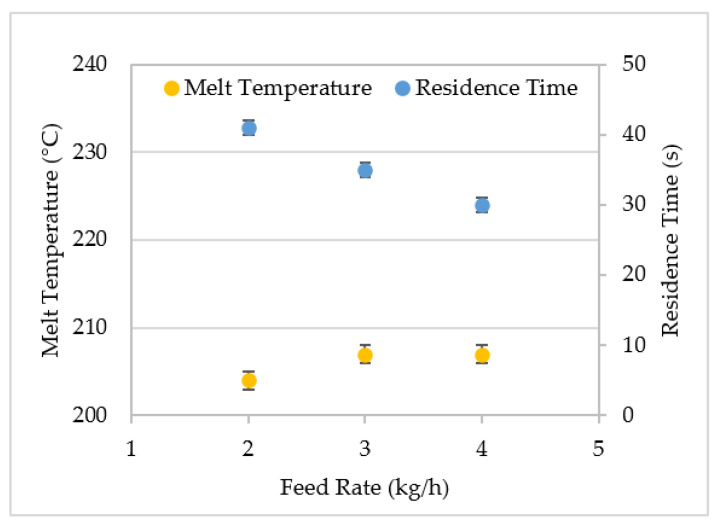
The effect of feed rate on the melt temperature and residence time for the QSE with screw configuration 2 and a screw speed of 400 rpm.

When the QSE was operated at a screw speed of 400 rpm, increasing the feed rate did not significantly increase the melt temperature. As illustrated in Figure 4, the melt temperature ranged from 204 °C to 207 °C. Although increasing the feed rate increases the filling of the screw channels and reduces the average shear rates, the QSE did not exhibit high levels of viscous dissipation. The findings are consistent with prior work [25], where increasing the feed rate from 1.68 kg/h to 3.2 kg/h produced a 7 °C increase in the melt temperature of polyethylene extruded at 500 rpm. In contrast, increasing the feed rate from 2 kg/h to 4 kg/h produced a linear decrease in residence time from 41 s to 30 s. The residence time (t_r_) could be modeled as t_r_ = 51.8 s–5.5 (Q_f_) where Q_f_ is the feed rate. The coefficient of determination was 0.99.

### 3.2. Rheology

**Figure 5:**
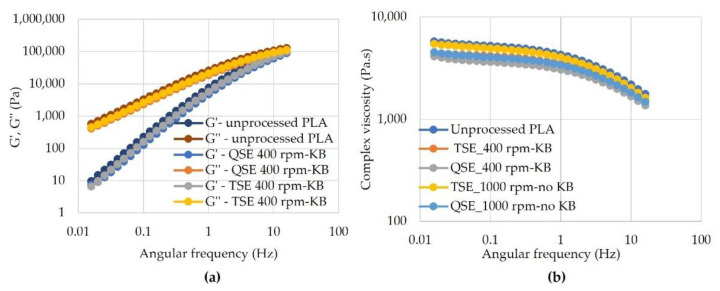
The effect of screw speed and screw configuration on rheology properties as a function of the angular frequency of polylactic acid processed using a TSE and QSE: (**a**) storage modulus (G′) and loss modulus (G″) and (**b**) complex viscosity. The feed rate was 2 kg/h.

Figure 5a displays the storage modulus (G′) and loss modulus (G″) as a function of angular frequency for PLA subjected to different processing conditions. The virgin (unprocessed) PLA and all processed PLA exhibited a predominant viscous behavior with G″ > G′. Similar results were reported by Cuadri and Martín-Alfonso, where G″ > G′ after poly (DL-lactide) was subjected to mixing times of 15, 30, and 60 min [35]. For this range of angular frequencies, crossover points where G′ = G″ were not observed; shifts in these crossover points indicate changes in the molecular weight and molecular weight distribution. Although the crossover points may have occurred at higher frequency ranges, their absence suggests that the molecular weight was relatively low, which is expected for condensation polymers such as PLA [36]. In general, the storage and loss moduli were lower for the processed samples and were higher for the TSE than the QSE. Although the data are not shown in Figure 5a, G′ and G″ were not significantly affected by changes in screw speed or screw configuration.

The complex viscosity–angular frequency curves are shown in Figure 5b. At low angular frequencies, the PLA exhibited a distinct Newtonian plateau. Rheological assessment of melted polymers performed at low frequencies was found to be one of the most efficient techniques for an indirect assessment of degradation changes of thermoplastic polymer materials during the process [20,35]. The processed PLA samples produced lower complex viscosities than the virgin PLA. The reductions in complex viscosity were greater in the QSE than in the TSE and increased for screw configurations with kneading blocks in the mixing zone. In prior work with twin screw extruders, a considerable reduction in the complex viscosity of processed materials was usually associated with random chain scission along the polymer molecules under thermo-oxidative conditions [20]. Thus, as the degree of degradation increases, so do the changes in the measured viscosities.

Table 2 presents zero-shear viscosities (η_o_) and the relaxation times (λ), which were determined when the viscosity–shear rate data were put into the Carreau–Yasuda model. The virgin PLA exhibited a zero-shear viscosity of 5672 Pa.s. Processing the PLA reduced the zero-shear viscosity, with the QSE and screw configurations with kneading blocks in the mixing zone producing greater reductions in zero-shear viscosity than the TSE and screw configurations with only convey elements, respectively. This behavior may have been due to the greater shear applied by the kneading blocks and the three intermeshing zones of the QSE, but it may also have been due to the increase in residence time. PLA extruded at 1000 rpm, however, showed higher zero-shear viscosities than when the screw speed was 400 pm. This significant reduction in degradation was attributed to the shorter extrusion residence time with the higher screw speed. For all processing configurations, the trend for residence time (t_r_) could be modeled as:

η_o_ = a + b t_r_ (4)

where the empirical constants a and b were 5750 Pa s and −41 Pa, respectively. The coefficient of determination was 0.85.

In addition, melt relaxation behavior can provide an indirect indication of changes in the molecular weight distribution of thermoplastic polymer materials [37]. The virgin PLA exhibited a relaxation time of about 76 ms, whereas the processed PLA showed lower relaxation times (Table 2). The relaxation time decreased with lower screw speeds, screw configurations with kneading blocks in the mixing zone, and the QSE, all of which increased the extruder residence time. The relaxation time (λ) for the processed PLA exhibited a linear relationship with residence time (t_r_):

λ = a + b t_r_ (5)

where the constant, a, was 93.8 ms and the slope, b, was −1.36. The coefficient of determination was 0.89. The reduction in relaxation time was attributed to the narrowing of the molecular weight distribution. Decreases in relaxation time had previously been observed in multiple grades of PLA processed using a TSE, with screw speeds of 50 rpm to 250 rpm [20] and polystyrene/polypropylene blends melt-compounded using a TSE [38]. For this work, the relaxation times for processed PLA in Equation (5) suggested a relaxation time for the virgin PLA (93.8 ms) that was greater than the measured relaxation time of 76 ms. This behavior indicates the relaxation time decreases more slowly with residence times that are shorter than those used in this study.

**Figure 6:**
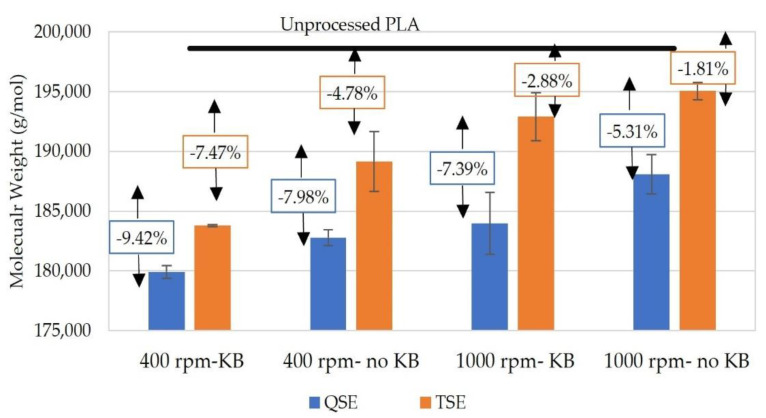
Weight-average molecular weight for polylactic acid processed using a TSE and a QSE. The arrows represent percentage reductions in molecular weight with respect to that of virgin PLA.

The weight-average molecular weights (M_w_) were calculated from the zero-shear viscosities in Table 2 using [39]:

(6) $$\eta_{o}=5.5\times 10^{-15}M_{w}^{3.4}$$

where the constants (5.5 × 10^−15^ and 3.4) had been determined for PLA. The weight-average molecular weights and their relationship to the weight-average molecular weights of the unprocessed PLA are presented in Figure 6. The calculated M_w_ for the unprocessed PLA (198,637 g/mol) was consistent with the molecular weight of polylactic acid (grade: 2003D) in material data mentioned by Shojaeiarani et al. [40]. In both screw configurations and screw speeds, the QSE exhibited lower M_w_ values than the TSE. The reductions in M_w_ ranged from 5.31% to 9.42% for the QSE, whereas they were 1.81% to 7.47% for the TSE. The presence of kneading blocks in the mixing zone and the use of lower screw speeds also reduced the weight-average molecular weight. Since the QSE produced only a minor increase in melt temperature, the degradation in molecular weight was attributed to the longer residence time that materials were exposed to shear during extrusion. Surprisingly, the significant rise in melt temperature (~25 °C) that occurred with the higher screw speed in the TSE did not substantially contribute to the reduction in molecular weight for both screw configurations. Overall, when the processing temperature was relatively low (180 °C), degradation of the molecular weight depended primarily on residence time during the process.

**Figure 7:**
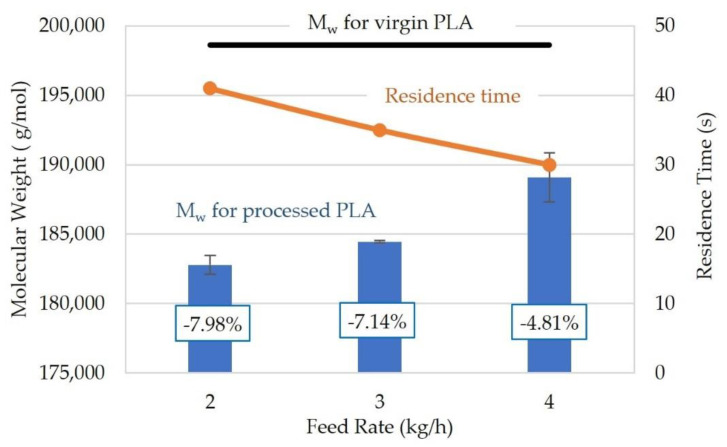
The weight-average molecular weight and residence time for polylactic acid processed using a QSE at a screw speed of 400 rpm and with screw configuration 2 (no KBs). The boxed values represent reductions in molecular weight with respect to the molecular weight of the virgin PLA.

To better understand the effect of residence time on the reduction in the weight-average molecular weight of PLA, residence times were controlled through feed rates for the QSE. As illustrated in Figure 7, residence time decreased linearly with increasing feed rate, but the calculated weight-average molecular weights increased in a non-linear fashion with feed rate. The reductions in weight-average molecular weight compared to the molecular weight on the virgin PLA also exhibited a non-linear relationship with feed rate. At a feed rate of 4 kg/h, the reduction in molecular weight was 4.81%, whereas the reductions were 7.14% and 7.98% for the slower feed rates of 3 and 2 kg/h, respectively. With a constant screw speed (400 rpm) and screw configuration, the higher feed rates reduced the extruder residence time and the average shear rate in the channels of the QSE. When the feed rate was 4 kg/h, the QSE residence time was 30 s; this residence time was similar to the 28 s recorded for the TSE with a 2 kg/h feed rate. The resultant molecular weights were also similar (~189,000 g/mol)—i.e., a 4.7–4.8% reduction compared to the molecular weight of the virgin PLA. In conclusion, these results also highlighted the impact of extruder residence time on the degradation of PLA.

### 3.3. Melt Flow Index

**Figure 8:**
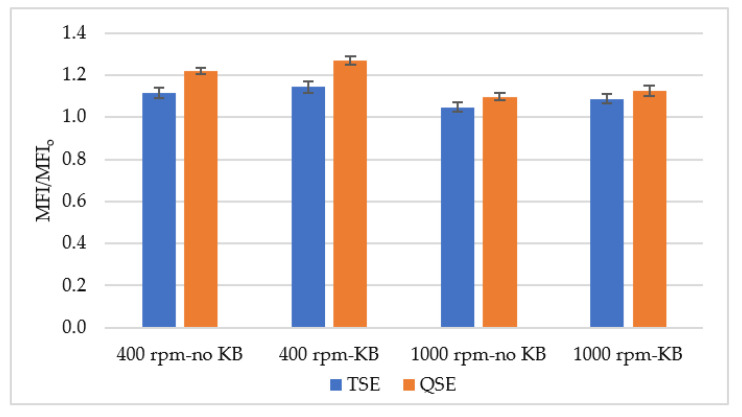
Melt flow index ratio (MFI/MFI_o_) for polylactic acid, processing the TSE and the QSE at two screw speeds and with two screw configurations.

The melt flow index (MFI) was measured to validate the rheological analysis. The melt flow index provides an indirect assessment of the polymer’s molecular weight based on flowability at relatively low shear rates [41]. Figure 8 presents the effect of screw speed and screw configuration on the relative melt flow index (MFI/MFI_o_). MFI/MFI_o_ values increased from 1.04 to 1.26, indicating degradation of PLA. The flowability of the processed PLA increased more with lower screw speeds, kneading blocks in the mixing zone, and the QSE. This behavior suggests that extruder residence time was controlling the degradation. Indeed, the MFI/MFI_o_ exhibited as a linear relationship with residence time (t_r_):

η_o_ = a + b t_r_ (7)

where the constant, a, was 1 (i.e., MFI_o_/MFI_o_) and the slope, b, was 0.0052 s^−1^. The coefficient of determination was 0.89. These findings were in a good agreement with the results from the rheological measurements discussed in Section 3.2.

### 3.4. Chemical Properties

**Figure 9:**
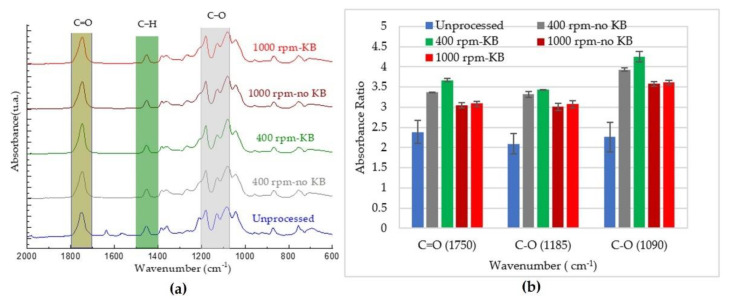
Effect of processing in the QSE on the chemical structure: (**a**) ATR-FTIR spectra and (**b**) absorbance ratios.

**Figure 10:**
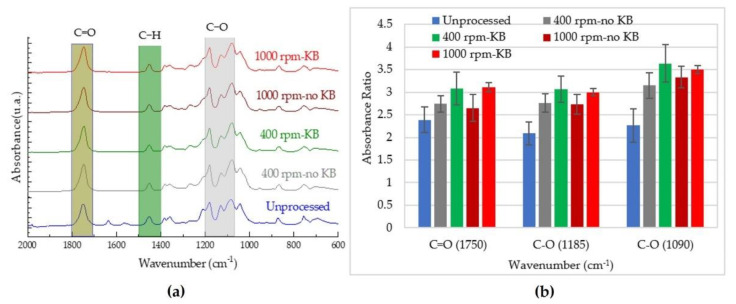
Effect of processing in the TSE on the chemical structure: (**a**) ATR-FTIR spectra and (**b**) absorbance ratios.

Changes in the rheological properties of PLA melts subjected to different processing conditions clearly exhibited degradation in the molecular weight of the polymer chains. Therefore, ATR-FTIR was used to assess the effects of these processing conditions on the chemical structure of PLA. Figure 9a and Figure 10a present summaries of the absorption spectra of unprocessed polylactide acid and the polylactic acid processed using different screw speeds and screw configurations in the QSE and TSE, respectively. The ATR-FTIR spectra were in the range of 2000 to 600 cm^−1^ and provided several insights. Firstly, upon processing PLA, the peak at 1630 cm^−1^ (which corresponds to H-O-H bending vibrations) was not visible in the infrared spectra. This result could have been due to thermal chain scission at the C–O bond [13]. Secondly, other peaks did not shift but showed changes in signal intensities, indicating that chain scission had occurred during processing. The most noticeable changes were at 1085 and 1183 cm^−1^, due to the asymmetric vibration of the ester group (C–O), and at 1750 cm^−1^, which is associated with carbonyl stretching (C=O) [22,42]. Thirdly, all processed PLA samples presented changes in their spectra in the 750 to 650 cm^−1^ region, which is attributed to changes in crystallinity [43].

As the changes in the hydroxyl band for the processed PLA were not significant, the degradation occurred mainly by chain scission. The peaks at 1090, 1185, and 1750 cm^−1^ were used to analyze chemical changes (i.e., chain scission) caused by processing. Increases in the carbonyl group peak have been attributed to the appearance of new carbonyl groups both in the middle and at the end of the molecules, which is a sign of reductions in molecular weight [44]. The absorbance ratios for these peaks were calculated from the heights of the peaks relative to the height of the reference peak. The reference peak was at 1455 cm^−1^ which is assigned to the asymmetric bending of the methyl group (CH_3_). These absorbance ratios are shown in Figure 9b and Figure 10b. In the unprocessed PLA, the absorbance ratios were 2.0–2.5. These ratios ranged from 2.5 to 3.5 for PLA processed using the TSE and about 3.0–4.1 for PLA extruded using the QSE, reflecting the greater degradation with QSE. In the QSE, the absorbance ratios were primarily influenced by the screw speed, with lower screw speeds exhibiting greater absorbance ratios. In contrast, the absorbance ratios for the TSEs also showed changes with screw configuration. In conclusion, the higher intensity of the absorbance bands correlated with the degradation of polylactic acid’s molecular weight at higher residence times.

### 3.5. Differential Scanning Calorimetry

The crystallization behavior of PLA can be influenced by its molecular weight [24]. Therefore, differential scanning calorimetry (DSC) was conducted to find out possible changes in the thermal behavior of PLA caused by the processing conditions in this study. Table 3 presents the glass transition temperatures (T_g_), melt temperatures (T_m_), and crystallinity values (χ_c_). In all processing conditions, glass transition temperatures were 57.5 °C to 58.5 °C and melt temperatures were approximately 143 °C to 146 °C. As multiple samples with the same processing conditions showed changes in T_g_ and T_m_ of 1 °C and 2 °C, respectively, these temperatures were not affected by extrusion conditions. The crystallinity varied from 0.16% to 1.37%, with multiple samples in the same processing conditions exhibiting variations of 0.06% to 0.14%. Therefore, only the QSE with KBs in zone 5 and a speed of 400 rpm produced samples with a significant change in crystallinity.

**Figure 11:**
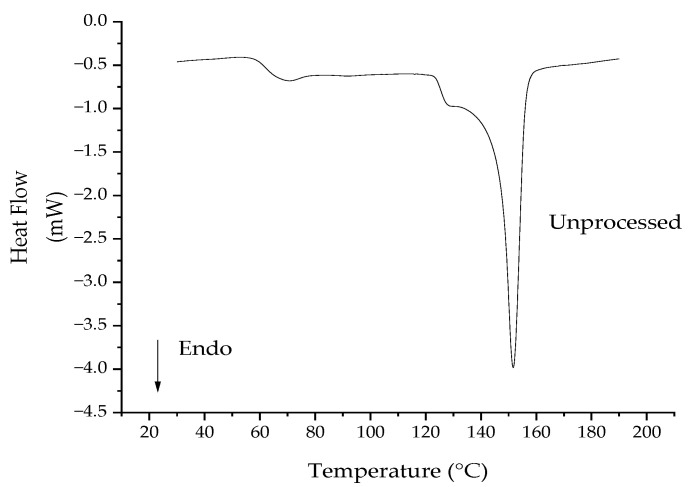
Heat flow temperature curve from the first heating cycle of the unprocessed polylactic acid in the DSC.

The low crystallinity values are better understood by examining the results from the virgin PLA pellets. During the first cycle, the glass transition temperature was 59 °C and the melt temperature was 146 °C. As the pellets had crystallized during manufacturing, there was no crystallization peak during the first heating ramp (Figure 11). The crystallinity of the unprocessed PLA was 34.45%. This value was similar to the 33.7% reported by Shojaeiarani et al. [40] for unprocessed PLA (grade: 2003D). During the second heating ramp, no melting peak was observed, indicating that recrystallization could not occur when the melt was cooled at 10 °C/min. The crystallinity during the second heating ramp was 0.16%. The cooling rate of 10 °C/min did not allow the PLA polymer chains to reorganize into crystalline regions because PLA has a low crystallization rate [45].

The crystallinities of the processed PLA were approximately 0%, because the absolute values of the cold crystallization and melting enthalpy are almost the same due to the slow crystallization rate of PLA and the relatively fast cooling rate (10 °C/min). The cooling rate was comparable to that used in other studies [20,35,41]. Mysiukiewicz et al. [20] also reported no changes in the crystallinity of multiple grades of reprocessed PLA in a TSE (similar results have been observed for high density polyethylene [35]). Oliveira et al. [41], however, found that the crystallinities were 34.4%, 46.1%, and 48.6%, respectively, for virgin PLA, PLA batch mixed for 5 min, and PLA batch mixed for 25 min. The melt temperatures decreased slightly. In conclusion, the extruder residence times in this work generally were not long enough to reduce the molecular weight sufficiently to change the crystallinity of PLA. The crystallinity was only 1.37% for PLA processed with the longest residence time of 41 s.

## 4. Conclusions

This work investigated the effect of two comparably sized compounding systems—a TSE and a QSE—on the degradation of polylactic acid (PLA). Compounding parameters included screw speed and screw configuration. Reductions in zero-shear viscosity and melt flow index indicated that the molecular weight of PLA decreased linearly with increasing residence time. Similarly, reductions in relaxation time suggested that narrowing of the molecular weight distribution decreased linearly with increasing residence time. Unexpectedly, the greater free volume and lower viscous dissipation of the QSE (compared to that of the TSE) were overridden by the longer residence time, as melt flowed across the four parallel screws. Factors that increased residence time, like the use of kneading blocks rather than conveying elements and the use of lower screw speeds, produced higher levels of degradation. Increased feed rates in the QSE reduced the residence time and, therefore, degradation of PLA. FTIR-ATR measurements confirmed that the major degradation mechanism was chain scission, and that greater chain scission occurred with longer residence times. The molecular weight reductions, however, were insufficient to produce major changes in the crystallinity of the processed PLA.

## Figures and Tables

**Table 1:** Processing conditions and equipment for the extrusion trials.

Parameter	Trial 1	Trial 2
Extruder(s)	TSE, QSE	QSE
Screw Configuration	1, 2	2
Screw Speed (rpm)	400, 1000	400
Feed Rate (kg/h)	2	2, 3, 4
Temperature (°C) Zone 1 to Zone 8 (Die)	165 to 180	165 to 180

**Table 2:** Zero-shear viscosity ($\eta_{o}$ ) and relaxation time (λ) for PLA processed using the TSE and QSE.

	Screw Speed (rpm)	Screw Configuration	η_o_ (Pa·s)	λ (ms)
Unprocessed PLA	-	-	5672 ± 35	76.1 ± 14.0
TSE	400	no KB	4806 ± 216	56.5 ± 22.8
		KB	4356 ± 57	51.8 ± 27.5
	1000	no KB	5332 ± 68	75.8 ± 1.7
		KB	5137 ± 182	66.8 ± 16.8
QSE	400	no KB	4276 ± 53	47.5 ± 9.2
		KB	4051 ± 42	35.4 ± 3.3
	1000	no KB	4713 ± 140	63.8 ± 11.4
		KB	4373 ± 210	52.8 ± 9.2

**Table 3:** Thermal properties for PLA processed using the TSE and QSE.

	Screw Speed (rpm)	Screw Configuration	T_g_ (°C)	T_m_ (°C)	χ_c_ (%)
Unprocessed (1st cycle)	-	-	58.9	145.8	34.45
Unprocessed (2nd cycle)	-	-	58.2	145.9	0.16
TSE	400	no KB	58.3	146.3	0.31
		KB	57.4	144.1	0.32
	1000	no KB	58.7	143.2	0.16
		KB	58.3	146.3	0.24
QSE	400	no KB	58.3	145.5	0.42
		KB	57.4	144.3	1.37
	1000	no KB	58.7	145.1	0.23
		KB	58.3	144.7	0.37

## Data Availability

The data presented in this study are available on request from the corresponding author.

## Abbreviations

**Abbreviation**	**Meaning**
ATR	attenuated total reflection
CH_3_	methyl group
C=O	carbonyl stretching
C–O–C	asymmetric vibration of the ester group
DSC	differential scanning calorimetry
FTIR	Fourier transform infrared radiation
G′	storage modulus45
G″	loss modulus
KB	kneading block in mixing zone (zone 5), refers to screw configuration
no-KB	without kneading block in mixing zone (zone 5), refers to screw configuration
MFI	melt flow index
PLA	polylactic acid
PLLA	poly (l-lactide)
QSE	quad screw extruder
Tg	glass transition temperature
Tm	melt temperature
TSE	twin screw extrusion
(χ_c_)	crystallinity
h_o_	zero shear viscosity
η*	complex viscosity
λ	relaxation time
